# Risk factors for treatment resistance among women with postpartum depression

**DOI:** 10.1192/j.eurpsy.2025.362

**Published:** 2025-08-26

**Authors:** Y. Chen, D. Lu

**Affiliations:** 1 Institute of Environmental Medicine, Karolinska Institutet, Stockholm, Sweden

## Abstract

**Introduction:**

Postpartum depression (PPD) impacts millions of new mothers worldwide, and the challenge of treatment resistance (TR) further hampers recovery prospects. The occurrence of TR in women with PPD and the risk factors for TR remain less studied. Identifying these factors is critical for the precise prediction of treatment responses, enabling tailored interventions and effective management of PPD.

**Objectives:**

This study aimed to determine the prevalence of TR and assess risk factors associated with TR in women with PPD in a nationwide population-based setting.

**Methods:**

We conducted a nationwide register-based cohort study of all women who gave birth during 2006-2021 in Sweden and were diagnosed with PPD up to 12 months postpartum. TR is defined as having ≥3 distinct antidepressant drugs, add-on medications, electroconvulsive therapy, or repetitive transcranial magnetic stimulation in one year after PPD diagnosis. Information on demographics, pregnancy characteristics and outcomes, comorbidities, and treatments were obtained from national registers. Potential risk factors in relation to TR were assessed using multivariable Poisson regression.

**Results:**

Out of 58 618 women with PPD (mean age 30.8, SD 5.3), 4 933 (8.5%) occurred treatment resistance. Younger age (<20 vs. 25-29y: risk ratio (RR) 1.28, 95% CI 1.07-1.52), lower educational level (<9 vs. >12y: 1.52, 1.39-1.67), lower family income level (lowest 20% vs. top 20%: 1.28, 1.16-1.40), smoking at early pregnancy (≥10 cigarettes/day vs. no smoking: 1.39, 1.19-1.62), prior physical comorbidities (Charlson comorbidity index ≥2 vs. 0: 1.40, 1.18-1.65), prior psychiatric disorders (RRs for specific types range: 1.54-6.04) were significantly associated with treatment resistance. In contrast, non-primiparous patients had a reduced risk of treatment resistance (vs. primiparous women, RRs for 2 parities: 0.74, 0.69-0.79; ≥3 parities: 0.87, 0.80-0.95). Maternal body mass index, snuff use, delivery method, pregnancy outcomes, and hypertensive or diabetic disorders did not predict treatment resistance.

**Conclusions:**

Treatment resistance in women with PD is common and is notably associated with specific demographic and clinical profiles. These findings highlight the need for personalized management strategies, particularly for identified high-risk groups.

**Disclosure of Interest:**

None Declared

